# A Study of "Medical Posture"

**Published:** 1883-12

**Authors:** John Kent Spender

**Affiliations:** Physician to the Mineral Water Hospital, Bath.


### A STUDY OF "MEDICAL POSTURE."

BY
JOHN KENT SPENDER, M.D. LOND.,
*Physician to the Mineral Water Hospital,*
*Bath.*

THE attention which has been given to surgical anatomy and surgical posture is not out of proportion to the scientific necessities of clinical surgery; but physicians have not devoted an equivalent share of study to anatomy and posture as an essential part of their own branch of therapeutic work. Until the publication of Dr. Sibson's splendid monograph, the subject of medical anatomy had never been formulated for systematic enquiry; certainly no proper instruction in it was offered to students of medicine thirty years ago. To regional surgery the utmost homage was paid, and faint was the hope of a candidate surviving the then mild ordeal of the College of Surgeons if he did not know the anatomical labyrinths of hernia, the physical signs of aneurysm, and the exact relations of vessels and nerves in all sorts of passages and cavities. But about the heart and the lungs and the liver—what was known of their size and precise situation in health, and of their subtle deviations in disease? To answer these questions there arose a race of physicians who published graphic tracings of the heart and its chambers as they lie behind the ribs; of the area of the lungs according as they are compressed or emphysematous; and of the position of the liver as it is pushed up or pushed down by intrinsic enlargement or extrinsic growths. It was a bold course, this diagrammatic help to clinical study; but it was in a high degree successful. The experiment evoked enthusiasm by its simplicity and obvious utility. I may liken it to the illustration of bare historical record by good maps and plans of battles; how vivid the narrative becomes, and how securely fastened in the memory! And so medical anatomy became a new science in the schools, and was gradually incorporated with the old learning.*

Now, *medical posture* is simply this—the application of an outward remedial system to a disturbed visceral anatomy, and to a disturbed visceral physiology. These conditions are not necessarily or universally bound up together. In the abdomen, for instance, the stomach and the colon are physiological miles apart; but their anatomical contiguity is such that the one may swell and touch the other, and a fistulous communication between them is not very uncommon. It is this *local nearness* of parts, irrespective of physiological relationship, which gives point and meaning to the subject of this paper. Embarrassed organs plead for more room to move in, and to do their duty. "More room" is the audible cry they utter when pain, and that distress which is nearly allied to pain, are the urgent signs. Whether the embarrassment be temporary or permanent is the business of medical anatomy to try and find out, and of medical posture to try and relieve. As physicians our province is to see that structures which are harmless neighbours when at peace, shall not hurt each other when there is patho-

* Drs. Fuller and Aitken were among the first physicians to embellish their writings with pictorial medical anatomy. *Geographical History* was the fruit of the teaching of the illustrious Dr. Arnold, developed in later times by those distinguished historians, Mr. E. A. Freeman and the late Mr. Green.

logical war. According as the body lies this way or that, heavy solid glands may be squeezed and displaced, hollow muscles may be hindered in their working, and vessels or ducts may be partially stopped up. A patient's own sensations usually tell some tale of the internal struggle, and may be a guide to the road which our remedial efforts should take. Here, then, is the neutral territory on which the doctor and the nurse may work in harmony, and where the nurse may justify the definition of her art as a "matter of quick observation and finished execution." Abernethy used to say that we heard much of the *tactus eruditus*, but very little of the *visus eruditus*, which should enable us to determine (by an almost unerring instinct) how far the physical surroundings of light, sound, touch, and posture may contribute to the well-being of any sufferer entrusted to our charge.

It is not necessary to be *ill* in order to experience all the benefits of medical posture. The gynecologist knows what a valuable resource he has in "position" when he desires to correct the many versions and perversions of which he is the reputed master. And there are conditions short of illness in which we welcome the aids of posture and rest. Mere fatigue dictates that balance of extensor and flexor muscles which provides the most ease to the tired organs of locomotion. Some people cannot sleep, however weary, unless the whole body be put into a flexile mould (so to speak), in which it may be shown that the contents of the thoracic and abdominal cavities enjoy the greatest freedom of action. We sometimes put our patients to bed not only for the sake of warmth and repose, but that they may have the liberty of finding out for themselves the attitude in which there is the most complete external and internal rest. But I press on *ad medias res*

and draw my illustrations from clinical practice, reminding my readers that I cannot do more than take hold of the fringe of a very large subject. The oracles wisely say that the transition from health to sickness is never abrupt, for "diseased states of the body are but shortcomings of the physiological standard" (Creighton); and again, "pathology is that branch of biology which concerns itself with disturbances of the normal life" (Huxley).

In the year 1865 I was asked to see a middle-aged man, a tradesman, who was said to be in a "fit." He was the patient of a medical neighbour who was out of town. On obeying the call I found the patient (quite unconscious) lying on a sofa, as nearly as possible flat upon his back, with a skin purplish blue, the body convulsed, and a noise of foaming râles in the chest. It was an instinctive act to turn the man gently upon his right side with the face looking downwards. Foamy saliva ran out of his mouth, the breathing became easier and the body warmer, and there was a gradual recovery without further anxiety. Now if I had been questioned on the spot it is doubtful if I could have rendered an exact reason for what was done. The man was very ill; the state of unconsciousness was increasing; the pulmonary embarrassment was becoming greater every moment, and the cyanosed skin showed that much unäerated blood could not get into the veins. "Something must be done" was the natural cry of those around, and the doctor was expected to do it. Nothing could be swallowed; there was no time for the action of enemata; and outward applications seemed to mock the gravity of the case. The man was slowly drowning in a pool of bronchial mucus. In ordinary health he had a slight bronchorrhœa, and the secretion soon gravitated into the smaller air-tubes when the usual efforts of expectoration were suspended. At this emergency "posture" stepped in as a static force, and brought relief to the oppressed functions when the dynamic powers were for the moment crushed. By the change of *decubitus* one lung was partially restored to use, and the respiratory muscles on one side were enabled to act with more advantage; while the altered position of the head allowed the watery sputa to find a more ready exit by the mouth.

It has been contended by Dr. Radcliffe that during an epileptic fit it is a great mistake to raise the head.* The head is raised on the supposition that the fit is caused by a rush of blood to the head, which is quite a mistake. During the fit the supply of arterial blood to the head is cut off by an arrest of the breathing; and on theoretical grounds, therefore, what ought to be done is to keep the head down rather than to raise it up. Take away the pillows, let the head fall over the edge of the bed; do everything which may help the blood towards the brain, and a succession of fits in an epileptic person may be cut short.†

If a very young child who has acute bronchitis be placed across the arms of a nurse with the face downwards, and kept in this position for hours at a time, it may make all the difference between gradual recovery and gradual asphyxia.

How many nice points there are in cardiac pathology which test the sagacity of the therapeutist. The living heart is a compendious expression of a number of forces,

* *Practitioner*, February, 1883.

† There are suggestions well worth studying in Sir H. Holland's *Medical Notes and Reflections* (what a rich but now little read book!) on the position of the head in certain affections of the brain (see chapter xxiii.). I may add that it would be difficult to formulate any posture of the head for the relief of the many varieties of headache.

vital and mechanical; and to favour the exercise of these forces under the stress of exhausting disease, so that they shall not come into conflict, is seldom an easy task. Take the matter of cardiac dilatation, and hear Dr. Russell, of Carlisle.* "In a debilitated heart only a small part of the contents of the left ventricle is expelled into the aorta; part regurgitates into the auricle, and the remainder is left in the ventricle. When a patient is recumbent it is easy to understand how a seriously weakened ventricle can carry on the circulation under conditions where a minimum of power suffices to pass on a little blood into the aorta, while the same weak effort relieves ventricular distension by regurgitation into a chamber occupying a posterior and therefore somewhat lower plane. Raise the patient, and the mechanical conditions are materially altered; the weight upon the aortic valves is increased, and more power is required to open them; at the same time the column of blood in the auricle falls into the partially-filled ventricle, and the chamber is over-distended; it is too weak to open the aortic cusps, or to lift the blood into the auricle, and death occurs by paralysis from over-distension." No words could portray with greater fidelity a source of danger not less in anæmia than in prolonged febrile affections.

Another indication for studying the posture of the thorax in disease is afforded by the theory lately advanced by Cohnheim, that œdema of the lungs is due to imperfect action of the left ventricle while the right is still functionally active. Obstruction to the pulmonary circulation must be very great under these circumstances, and a local dropsy must follow if the obstruction be sufficiently long and complete. During perfect health

* *Brit. Med. Journal*, December 30th, 1882.

the heart and the lungs dwell in a sort of international comity, each helping the other, each depending on the other, but neither can do the other's work. Both are, however, *very jealous of trespass;* and when that trespass consists of increased bulk pushing beyond its natural boundary and occupying space at the expense of its neighbour, the invasion is resented, belligerent signals are hoisted, and auscultation and percussion reveal what a strife is going on. The struggle for mastery issues in vascular and neurotic disorder, which may be the prelude of serious and incurable disease. Now if we cannot prevent this disease being developed, should we not study how to give *more room* to those organs which are oppressed and fighting against overwhelming odds? Should we not alleviate suffering by doing so? If we fail to execute this obvious mandate, the patient will take the duty out of our hands, and try and discover for himself how to mitigate his distress.

*Orthopnœa* is commonly called a symptom, but it is really a therapeutic posture. It is a behaviour of the body instinctively adopted for certain ends. So far as the upper part of the body is concerned, orthopnœa is an adjustment of organs for soothing the chronic anguish of pressure on nerves and vessels; for letting the air get more easily to the blood, or the blood more easily to the air; and for lessening the upward stress of abdominal viscera on heart and lungs.* Whenever the pulse and respiration ratio is disturbed, if the pulse be quiet and the respiration high, then the thoracic space must be dimin-

* Looked at as a remedial symptom, orthopnœa has grave disadvantages, as Dr. Fagge has pointed out (Reynolds' *System of Medicine*, vol. iv., p. 670). It prevents sleep, and keeps the lower limbs at right-angles with the trunk, so that œdema of the legs is increased. It fatigues the lumbar muscles and makes the back ache.

ished by a slow malady, such as a solid tissue or a morbid growth, a pleuritic effusion, or an excess of blood in the pulmonary circulation. For any of these chronic conditions posture is a therapeutic agent of the highest value; a modification of orthopnœa it may be, its form being generally the sloping chest and the raised head; but when the pulse is high and the respiration comparatively infrequent, some acute or febrile process must be going on in which postural aids are of little significance. I have manuscript notes of an unpublished lecture by the late Dr. Todd, in which he dwells on the necessity of helping the right heart *by position of the body* when it is embarrassed by whooping cough and asthma, or by other pulmonary difficulties creating a barrier to the easy transit of the blood. The rational therapeutics of cyanosis would include a leaning to the right side when the body is recumbent; and for palpitation of the heart unconnected with organic disease, it has been recommended to lower the head so as to produce a mechanical congestion of the upper half of the body.

The treatment of the three forms of stertor—palatine, pharyngeal, and mucous, has been explained and illustrated by Dr. Bowles.* In a case related, stertor ceased when the patient was placed upon her side, and a gradual improvement followed; stertor returned and death seemed impending when she was placed upon the opposite side; then again there was instant relief when the former position was renewed, and a return to consciousness was coincident with the cessation of the stertor. It is argued that the varieties of stertor are the immediate results of a local mechanical condition, which may always and at

* Dr. Bowles' original paper was published in the 48th volume of the *Medico-Chirurgical Transactions.*

once be changed; and that in the so-called apoplectic state the paralysed side should be always placed downwards, for the lung of the paralysed side becomes loaded with a mucous fluid, which gravitates to the opposite lung whenever the patient is placed with the sound side downwards. In its passage across the trachea the mucus becomes churned into foam by the ingoing air, causing stertor, great dyspnœa, and possibly death. These principles apply to all conditions in which mucus or fluid exists in either or both lungs; and Dr. Bowles shows that by keeping these facts in mind we may relieve many cases of hæmoptysis and capillary bronchitis.*

During the inhalation of any anæsthetic gas or vapour, the head of a patient should be kept on a level with the body, or on a gradually inclined plane; for if the body be doubled forwards by too great a number of pillows under the head, "curves are made in the windpipe." Dr. Eben. Watson has been lately questioning the propriety of inverting patients in apparent death from the inhalation of chloroform and from drowning, and on good physiological grounds; while Dr. Silvester has followed the same line of argument in teaching that in the treatment of the apparently dead the most suitable position is that of lying on the back, the body inclining a little from the feet upwards, the shoulders and head slightly raised and supported on a firm cushion. This position is favourable for the relief of congestion of the heart and the head, and both sides of the chest are free to expand.†

It goes almost without saying that the therapeutics of

* A medicine opportunely administered may do indirect good by making a natural posture comfortable. Thus after the action of ipecacuanha as an emetic (a "good remedy out of fashion," says Dr. Hare) in capillary bronchitis, a patient may lie moderately low in bed and get some sleep.

† *Lancet*, March 10th and May 19th, 1883.

the abdomen is to a large extent a question of posture. I do not, therefore, recapitulate what is familiar to every student, and ordered as a matter of routine by careful practitioners. But there are one or two points which are not so generally recognised. In treating sea-sickness the body should be put on an inclined plane with the head quite low. Keep as much blood in the brain as possible. More effective in other cases is firm pressure over the stomach by coiling the body around a bolster. In treating an assumed ulcer of the stomach it is imperative that the patient be kept in the recumbent posture in order that the stomach walls may be in a state of rest.* Some forms of persistent vomiting are controlled only by keeping the body in a semi-recumbent position; and the same safe and easy remedy helps to cure gastric and duodenal dyspepsia.

When intussusception is suspected as the cause of an obstruction of the bowels, an enema should be administered while the child is put upon its face, and the nates raised.

In many troubles of the pelvic viscera what comfort is afforded by the simple expedient of lifting the foot of a couch five or six inches. A great solace this is to many a weary woman! The involuntary nocturnal micturition of children, which has perhaps defied a well-planned scheme of physiological diet and drugs, may yield at once if the end of the cot be elevated so as to make the feet of the little patient slightly higher than the head.

In sundry kinds of external neuralgia, a consideration of posture may relieve pain by liberating the painful part

* This recommendation was given by Dr. Brinton in his classical book on *Diseases of the Stomach*, so long ago as 1859, but is not always properly attended to.

from being dragged or squeezed. In the treatment of sciatica, the muscles are relaxed, and the body naturally turns on the painless side; and the arm ought to be supported when its weight would cause or increase pain in the cervical or brachial plexus of nerves.

In the use of our thermal Waters, position requires to be sometimes thought of, and we order the *reclining* bath when by reason of age or infirmity the patient must recline during the process of bathing. And speaking of age and infirmity, may there not be too much rest, or too much of the postures which imply a great deal of rest? Wise advice is that given by Cicero in the *De Senectute*, "*Quod est, eo decet uti;*" of which a free translation would be: "As long as you can walk about, do not ride too often in Bath chairs!"

Finally and conversely, we now and then learn from a person's posture what his disease is or may be. This deductive method is not unknown in the more purely medicinal branch of therapeutics: for even an expert like Dr. Buzzard is obliged to confess that he is not always able to diagnose the syphilitic etiology of a given cerebro-spinal lesion, apart from the fact that iodide of potassium has cured or, at least, much relieved it.

The sketch which has been now attempted of the doctrine of medical posture is necessarily very imperfect. Within the allotted space of a periodical it is not possible to present more than a fragment of a large subject; or, to speak more appropriately, more than bare outlines which may be filled in by a more extended observation and a riper experience. The refinements of therapeutic art are not bound by the discoveries of *Materia Medica* or by the manipulations of the pharmaceutist. Amorphous and almost chaotic as some groups of clinical phenomena appear to be, and seemingly beyond the most far-reaching generalization, yet they may contain an element of static disturbance which can be put right by a shift of position, a new plane, a hard pillow, or even by a fresh adjustment of clothes. No trifle is beneath the attention of the doctor; his drugs and his hygiene may operate sooner and with more completeness when every detail of his work is under his eye, and when with the sagacity of good generalship he esteems nothing as unworthy of his command. Nothing is to be despised which helps us in the battle against weakness and disease, and which keeps in order a little longer that LIFE which Bichat called a "collection of functions designed to resist death."

